# Allo-HSCT with TBI-based preconditioning for hepatosplenic T-cell lymphoma: two case reports and systematic review of literature

**DOI:** 10.3389/fonc.2024.1345464

**Published:** 2024-01-29

**Authors:** Can Chen, Fan Yang, Peiwen Miu, Pengfei Shi, Shenxian Qian

**Affiliations:** ^1^ Department Of Hematology, Hangzhou First People’s Hospital, Hangzhou, China; ^2^ Fourth Clinical College, Zhejiang Chinese Medical University, Hangzhou, China

**Keywords:** hepatosplenic T cell lymphoma, allogeneic hematopoietic stem cell transplantation, total body irradiation, preconditioning, prognosis, systematic review

## Abstract

Hepatosplenic T cell lymphoma (HSTCL) is a particularly difficult-to-treat form of lymphoma, with many patients exhibiting primary resistance to chemotherapy. At present, no effective strategy for treating relapsed and refractory HSTCL has been established, with treatment being hampered by questions of how best to overcome chemoresistance to allow patients to attain more durable therapeutic benefits. While there have been marked advances in immunotherapy, allogeneic hematopoietic stem cell transplantation (allo-HSCT) remains one of the primary approaches to curing HSTCL. Of patients who undergo immunochemotherapeutic treatment, many are resistant to conventional chemotherapeutic drugs yet remain sensitive to radiotherapy. We selected to employ a transplant pretreatment regimen consisting of total -body irradiation (TBI) and administered this regimen to two patients with HSTCL. Both patients achieved complete remission (CR) after transplantation, demonstrating extended periods without disease recurrence. We systematic reviewed previously published instances involving allo-HSCT in patients with HSTCL. We have found a total of 67 patients who have received allo-HSCT. In general, age<45 and the status of CR at HSCT may have a more favorable prognosis. Although the impact of TBI on prognosis was not found to be substantial, patients in the TBI group had higher 3-year overall survival (66.7% vs. 71.1%) and 5-year overall survival (58.4% vs. 71.1%) compared to patients in the non-TBI group. In addition, the relapse rate of the TBI group is approximately half that of the non-TBI group. This regimen is well tolerated and associated with low recurrence rates or complications, suggesting that it represents a viable pretreatment regimen for young HSTCL patients undergoing allogeneic HSCT.

## Introduction

Hepatosplenic T cell lymphoma (HSTCL) is a rare and highly aggressive form of peripheral T cell lymphoma that is most commonly diagnosed in younger males, resulting in a disease characterized by systemic symptoms, thrombocytopenia, and hepatosplenomegaly without corresponding lymphadenopathy (1). Pathological examination of affected patients often revealed neoplastic cells in the splenic red pulp with infiltration of the hepatic, splenic, and bone marrow sinusoids (2). While γδ T cell receptor (TCR) expression is observed in most cases, some HSTCL cases exhibit αβ TCR expression, with both subtypes exhibiting a similar clinical course such that αβ HSTCL is generally regarded as an immuno-phenotypic variant (3). Approximately 20% of HSTCL cases develop in patients with a history of immunosuppression, and this disease is not related to infection with Epstein-Barr virus (EBV), in contrast with other forms of immune-mediated lymphoma (4, 5).

HSTCL exhibits a very aggressive clinical course, with poor chemotherapy response rates, an extremely low 7% 5-year survival rate, and a median overall survival duration of just 10 months across age groups (6). While there have been a few case reports documenting long-term survivors diagnosed with HSTCL, no standardized strategy for treating affected patients has yet been established (7). Most published data comprise case reports or case series in which CHOP (cyclophosphamide/doxorubicin/vincristine/prednisone)-based regimens have been linked to poor long-term outcomes (6). Even among patients who achieve complete remission (CR) after induction, the median overall survival duration tends to be short as the duration of disease remission tends to be short (8). The only curative treatment option for HSTCL patients is allogeneic hematopoietic stem cell transplantation (allo-HSCT). This report describes two cases of patients diagnosed with HSTCL who underwent allo-HSCT with a TBI combined precondition regimen. In addition, a systematic review of literature on adults with HSTCL who underwent allo-HSCT is also provided.

## Case presentation

### Patient 1

**Figure 1:**
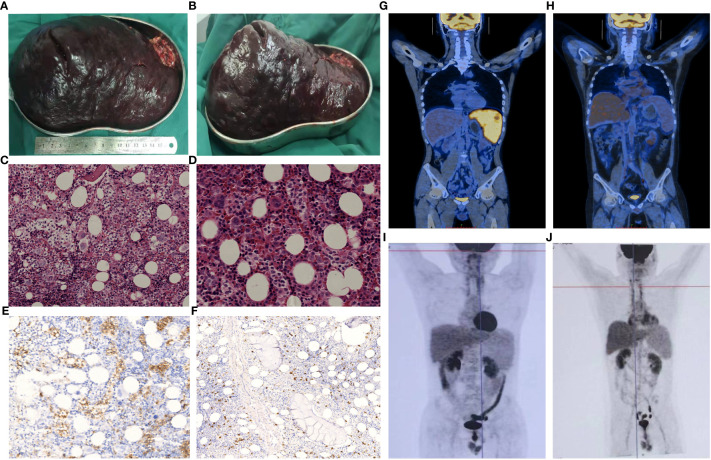
Clinicopathological features of the two patients. In patient 1, the splenectomy specimen revealed significant splenic enlargement, as demonstrated in images **(A, B)**. Bone marrow histology of patient 2, stained with HE **(C)**, x200; **(D)**, x400), exhibited infiltration of lymphoma cells. These cells appeared as small to intermediately sized lymphoid cells within the medullary sinus, displaying slight irregularities, loose chromatin, and inconspicuous nucleoli. Immunostaining indicated that these cells were positive for CD3 **(E)**, x400) and negative for CD5 **(F)**, x200). A PET-CT scan performed on patient 1 before **(G)**, 2/1/2021) and after **(H)**, 1/26/2022) allo-HSCT demonstrated complete remission. Post allo-HSCT, patient 2 showed no abnormalities on PET-CT scans conducted on 5/13/2021 **(I)** and 1/26/2022 **(J)**.

In 2020, a 29-year-old man with ecchymosis of both lower limbs showed up with no obvious explanation. Despite corticosteroids, the patient continued experiencing splenomegaly and increasing thrombocytopenia, increasing the possibility of an ITP diagnosis. In February 2021, the patient reported to our hospital. At that time, physical examination revealed that the lower pole of the spleen was located two finger widths above the umbilicus and was palpable below the left costal margin. After a thorough assessment (**Table 1**), a splenectomy was performed, ensuring that there were no contraindications. Significant splenic enlargement was observed during the surgery (**Figures 1A, B**). The histological findings revealed cells of moderate size with oval-shaped nuclei, exhibiting abnormalities in their nuclear structure and small nucleoli. Immunohistochemistry results of spleen revealed these cells were CD2-, CD3+, CD5-, CD7+, CD20- PAX5-, CD79α-, CD23-, TIA-1+, CD56-, granzyme B-, CD4-, CD8-, CD30-, CD138-, ALK-, Ki-67 + 60%. Cells were positive for TCRγ rearrangement. The splenic flow cytometry analysis was performed, which demonstrated abnormal T cells accounted for ~22.94% of total cells, and these cells were CD7++, CD16++, CD33-, CD13-, CD38-, CD117-, CD19-, CD34-, HLA-DR dim, CD56-, CD5-, CD 5-, CD25-, CD3++, CD99dim, CD2 minimal expression, CD4++, CD8-, TCRαβ-, TCRγδ+, CD335-, CD28dim, CD24, CD57-, CD94+, CD337-, CD158a,h-, CD158f-, CD158e1/e2-, CD158b1/b2j+, CD158i (46.00%). Therefore, a definitive diagnosis of stage IE Hepatosplenic T-cell Lymphoma (HSTCL) was made.

**Table 1:** Clinical examination results of two patients.

Examination	Result of Patient 1	Result of Patient 2
Routine blood count	Platelet 27x10^9^/L	White blood cell count 0.7x10^9^/L, platelet count 47x10^9^/L, and hemoglobin 113 g/L.
Chest/abdominal CT	Splenomegaly	Splenomegaly with uneven density, increased liver volume, and limited effusion of the left chest and abdominopelvic cavity
PET-CT	Splenomegaly, with a SUVmax of 8.5.	Not performed.
Liver and kidney function	Lactate dehydrogenase level was elevated (368 U/L, normal range: 50-240 U/L) without other abnormal results.	Normal.
B ultrasonography	Splenic area of 14.5 x 6 cm.	Liver was normally sized, while the spleen was enlarged (23 x 12 cm) with full morphology, smooth contours, and uneven local parenchymal echo at the lower pole.
Bone marrow puncture	Clear evidence of hyperplasia, with 850 megakaryocytes in the entire film, primarily of the naïve and granular types and exhibiting poor functionality.	Clear hyperplasia, with 24.0% lymphocytes, some loose nuclear chromatin, and villi or pseudo-like processes at the margins of small lymphocytes.
Bone marrow flow cytometry	Normal.	T lymphocytes that were CD45+, CD3+, CD5-in accounted for ~11.2% of nucleated cells, and these cells were found to express CD2, CD3, CD7, CD45RO, and TCR γδ but not CD4, CD5, CD8, CD16, CD30, CD57, TCR αβ, or CD45RA.
Bone marrow biopsy	Consistent with the hypohyperplasia of bone marrow hematopoietic tissue and 10-20 megakaryocytes per low-powered field.	Presence of small- to intermediately-sized lymphoid cells in the medullary sinus, with slight irregularities, loose chromatin, and inconspicuous nucleoli.
Immunohistochemistry results of Bone marrow	Normal.	Lymphoid cells: MPO-, TDT-, CD34-, CD117-, CD10-, CD3+, CD5-, CD2 weak+, CD7+, CD4-, CD8-, CD43 weak+, CD20---, CD30--, CD56--, granzyme B--, TIA-1++, Ki-67+ 10-15%+, CD42b megakaryocytes+, Gomori 1+. The T cell receptor (TCR) rearrangement results showed TCRGA+, TCRGB+, and TCRD+.
Biopsy result of spleen	Cells were of intermediate size with ovoid nuclei, nuclear irregularities, and small nucleoli.	Not performed.
Immunohistochemistry results of spleen	CD2-, CD3+, CD5-, CD7+, CD20- PAX5-, CD79α-, CD23-, TIA-1+, CD56-, granzyme B-, CD4-, CD8-, CD30-, CD138-, ALK-, Ki-67+ 60%. Cells were positive for TCRγ rearrangement.	Not performed.
Splenic flow cytometry	Abnormal T cells accounted for ~22.94% of total cells, and these cells were CD7++, CD16++, CD33-, CD13-, CD38-, CD117-, CD19-, CD34-, HLA-DR dim, CD56-, CD5-, CD 5-, CD25-, CD3++, CD99dim, CD2 minimal expression, CD4++, CD8-, TCRαβ-, TCRγδ+, CD335-, CD28dim, CD24, CD57-, CD94+, CD337-, CD158a,h-, CD158f-, CD158e1/e2-, CD158b1/b2j+, CD158i (46.00%).	Not performed.

**Figure 2:**
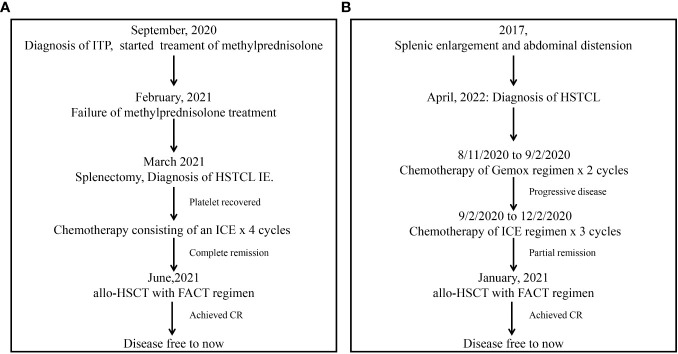
Clinical course of patients 1 **(A)** and 2 **(B)**.

At approximately 3 weeks after surgery, the patient’s platelet levels had recovered to within the normal range. Chemotherapy consisting of an ICE regimen (ifosfamide 2.7 g d1-3, etoposide 0.15 g d1-3. carboplatin 0.5 g d2) was administered on 2/23/2021, 3/15/2021, 4/12/2021, and 5/12/2021, and after treatment PET-CT results were negative and the patient was considered to have achieved CR. As HSCTL is an aggressive disease with a poor prognosis, it was recommended that the patient undergo allo-HSCT. A FACT conditioning regimen was initiated on 6/18/2021 consisting of TBI (6 Gy, d -10), Fludarabine (150 mg/m^2^ in 5 days, d -9 to -6), Cytarabine (10 g/m^2^ in 5 days, d -9 to -5), Cyclophosphamide (2 g/m^2^ in 2 days, d -4 to -3), T -cell depletion with anti-human thymoglobulin (10 mg/kg in 4 days, d -4 to d -1). GVHD prophylaxis consisted of cyclosporine, mycophenolate mofetil, and low -dose methotrexate (MTX). Between 6/29/2021 and 6/30/2021, haplotypic hematopoietic stem cells were transfused (HLA matched 6/12 with blood type donor A+ for recipient A+, with mononuclear cell (MNC) counts of 8.08x10^8^/kg and CD34+ cell count of 2.54x10^6^/kg. On 6/28/2021, an auxiliary transfusion of cord blood was performed (HLA 7/10, O+ for A+), with a total nucleated cell (TNC) count of 2.2x10^7^/kg and a CD34+ cell count of 1.34x10^5^/kg. Leukocyte and platelet were engrafted on days +10 and +11, perspectively. Acyclovir was used as a precaution to prevent cytomegalovirus (CMV) activation. On day +46, the patient’s CMV DNA levels had risen to 7.82x10^3^ copies/mL but improved with ganciclovir antiviral treatment. On day +85, the patient developed nausea and vomiting after excluding CMV or EBV infection. This was considered an instance of grade I/II gastrointestinal graft-versus-host response. Glucocorticoid and cyclosporine were used for anti-rejection treatment and were effective. On day +443, the patient developed herpes zoster infection, which improved with valaciclovir antiviral therapy. PET-CT scans (**Figures 1G, H**) revealed a substantially reduced number of lesions compared to the prior scans. Currently, the patient remains free from disease and has survived for 732 days following the transplantation procedure. The patient’s overall health was good, as shown in **Figure 2A**.

### Patient 2

A 30-year-old male received medical treatment at our hospital in July 2020 due to a 3-year history of an enlarged spleen and abdominal swelling without any other associated symptoms. Recently, he has been suffering from inexplicable fatigue, decreased appetite, increased abdominal distension, and a significant weight loss of approximately 10 kg in just 3 months. Systemic examinations were performed, as indicated in **Table 1**. Bone marrow biopsy revealed apparent hyperplasia, with 24.0% lymphocytes, some loose nuclear chromatin, and villi or pseudo-like processes at the margins of small lymphocytes. Bone marrow flow cytometry revealed T lymphocytes that were CD45+, CD3+, CD5-in accounted for ~11.2% of nucleated cells, and these cells were found to express CD2, CD3, CD7, CD45RO, and TCR γδ but not CD4, CD5, CD8, CD16, CD30, CD57, TCR αβ, or CD45RA. Bone marrow biopsy revealed small- to intermediately-sized lymphoid cells in the medullary sinus, with slight irregularities, loose chromatin, and inconspicuous nucleoli. Immunohistochemistry results showed that the lymphoid cells were MPO-, TDT-, CD34-, CD117-, CD10-, CD3+, CD5-, CD2 weak+, CD7+, CD4-, CD8-, CD43 weak+, CD20—, CD30–, CD56–, granzyme B–, TIA-1++, Ki-67 + 10-15%+, CD42b megakaryocytes+, Gomori 1+. The T cell receptor (TCR) rearrangement results showed TCRGA+, TCRGB+, and TCRD+. The diagnosis of HSTCL was conclusively confirmed through a bone marrow biopsy (**Figures 1C-F**). The PET-CT scan was excluded because of its excessive cost. The diagnosis of HSTCL was considered based on these data.

After excluding contraindications, a P-Gemox chemotherapeutic regimen (gemcitabine needle [1.6 g d1, oxaliplatin [0.16 g d1], and Pegaspargase [3750 U d2]) was initiated on 8/11/2020. A P-Gemox regimen was repeated beginning on 9/2/2020, and after treatment, B ultrasonography revealed splenomegaly with localized infarction. In addition, the patient experienced a fever associated with the tumor, suggesting that the earlier chemotherapy was ineffective. An ICE chemotherapy regimen was administered on 10/16/2020, 11/14/2020, and 12/5/2020 consisting of etoposide (0.16 g d1-3), ifosfamide (2 g d1-3), and carboplatin (450 mg d1). After treatment, the spleen shrank, consistent with partial remission.

On 1/11/2021, allo-HSCT treatment was initiated using a FACT conditioning regimen. The drugs used for GVHD prophylaxis include cyclosporine, mycophenolate mofetil, and low-dose MTX. Between 1/21/2021 and 1/22/2021, haplotypic hematopoietic stem cells were transfused (HLA matched 6/12, blood type donor O+ for recipient O+) with MNC counts of 10.72x10^8^/kg and a CD34+ cell count of 7.63x10^6^/kg. Leukocytes and platelets were engrafted on days +16. Acyclovir was used to protect against CMV infection after transplantation. On day +59, blood tests for CMV DNA revealed a titer of 3.10x10^3^ copies/mL, and sodium foscarnet was provided for antiviral therapy. Due to the high risk of relapse, Donor lymphocyte infusion (DLI) was treated at day +84 and day +140.

On day +119, B ultrasonography of the liver revealed enlargement and echoic changes with an increase in splenic volume (18.1 x 6.9 cm), and the lower splenic pole was located two finger-widths above the umbilicus. The PET-CT scan (**Figure 1I**) showed no abnormal glucose uptake by the spleen. On day +182, the patient exhibited skin and oral mucosa grade I GVHD, which improved with glucocorticoid therapy. On day +243, Alanine aminotransferase levels were significantly elevated and improved following hormone administration and hepatoprotective treatment. On day +431, B ultrasonography suggested the presence of multiple hypoechoic nodules in the bilateral axilla, which were more prominent on the right side, being about 4.1x2.2x2.8 cm in size. Substantial splenic enlargement was noted (~4.8 cm thick). An axillary lymph node biopsy was performed, and pathology did not reveal any evidence of lymphoma. Repeat PET-CT (**Figure 1J**) scans were negative. The patient remains disease-free at day 902 after transplant (**Figure 2B**).

### Systematic review

In order to provide a complete overview of the clinical features and outlook for patients with HSTCL who received HSCT, we systematically assessed the available literature. The included **Supplementary Table S1** provides a comprehensive overview of our search methodology, with retrieval and outcome analyses being conducted by two independent researchers. Due to the primary statistical focus on the prognosis of transplant patients, material that did not include precise survival data, especially overall survival (OS), was excluded. We incorporated 30 research papers encompassing 67 patients (including two participants from this study). In cases where articles or attachments did not expressly include precise clinical details, we used the terms ‘Not Available’ or ‘Not Defined’ to indicate the absence of data. This was accomplished to avoid any later statistical analysis in survival assessments.

Among the individuals who underwent transplants were 41 males and 20 females. The majority of them, precisely 44 out of 61, were under the age of 45. 9 individuals suffered from having spleens removed, and 58 patients were classified as stage IV. During the transplantation process, 25 patients were found to be in a state of complete remission, 26 patients were in a state of partial remission, and 11 patients were not. The donor selection process consisted of 17 instances involving sibling donors, 7 cases involving haploidentical transplants, 18 cases involving unrelated donors, and 4 cases involving cord blood. The origins of the transplants consisted of 12 cases of bone marrow, 16 cases of peripheral blood stem cells, and 4 cases of cord blood. The transplant intensity was classified as MAC in 25 cases, RIC in 11 cases, and NMAC in 1. A total of 33 patients received transplant conditioning regimens involving TBI, with the majority being under the age of 45 (29 out of 33, P=0.002). Fourteen of these cases reported the dose and method of TBI application. Seven received >6GY, and 7 received ≤6GY. The majority (10/12) underwent fractionated radiation therapy. The predominant protocols implemented for GVHD prevention were CSA or FK506, and the GVHD incidence was 72.9%(27/37) (**Table 2**).

**Table 2:** Clinical characteristics of patients reviewed.

Clinical characteristics		Patients Number*
Gender	Male	41
	Female	20
Age	≥ 45	17
	<45	44
Stage	I	2
	III	5
	IV	58
Prior splenectomy	Yes	9
	NO	32
Status at HSCT	CR	25
	PR	26
	NR	11
Donor Source	Sibling	17
	Haplo	7
	MUD	18
	UBC	4
Graft Source	BM	19
	PB	16
	UCB	4
Conditioning intensity	MAC	24
	RIC	11
	NMAC	1
Conditioning include TBI	Yes	33
	No	20
TBI dose	≤6GY	7
	>6GY	7
TBI fraction	Yes	10
	No	2
GVHD prophylaxis	CSA+MMF+MTX	7
	CSA+MTX	2
	CSA+PTCY	2
	FK506	2
	FK506+MMF	1
	FK506+MMF+PTCY	1
	FK506+MMF+MTX	1
GVHD	aGVHD	18
	cGVHD	4
	aGVHD+cGVHD	3
	No GVHD	10
	Not defined	2
Relapse after HSCT	Yes	11
	NO	48
Status of patients	Alive	45
	Died	22
Main course of death	TRM	3
	Primary Disease	4
	Infection	1
	GVHD	3
	GVHD and Infection	3
	GVHD and VOD	1
	VOD	1
	Others	3

*Each group includes only the patients for whom data is available.

CR, complete remission; PR, partial remission; NR, no remission; TBI, total body irradiation; Haplo, haploidentical; MUD, matched unrelated donor; UCB, unrelated core blood; MAC, myeloablative conditioning; RIC, reduced intensity conditioning; NMAC, non-myeloablative; BM, bone marrow; PB, peripheral blood; CSA, Cyclosporine; MMF, mycophenolate mofetil; MTX, methotrexate; PTCY, post-transplantation cyclophosphamide; FK506, tacrolimus; GVHD, graft versus host disease; TRM, treatment relate mortality; VOD, veno-occlusive disease.

Eleven patients suffered a recurrence, accounting for 18.6% of the total (11/59). Before transplantation, patients who achieved complete remission (CR) appeared to have a decreased relapse rate (4.7% vs 25.7%, P=0.072). The number of recorded deaths was 22, resulting in a mortality rate of 32.8% (22 out of 67). Patients under 45 exhibited a reduced mortality rate (27.2% vs 52.9%, P=0.076). In addition, patients who achieved CR before transplantation had a significantly lower mortality rate compared to those who did not (16% vs 45.9%, P=0.027). Out of the 19 patients whose reasons for death were recorded, only 4 died as a result of disease progression (**Tables 2**, **3**). No indicators were identified as affecting GVHD (**Supplementary Table S2**).

**Table 3:** Clinical factors associated with survival status and relapse.

Clinical Chracteristics*		Status		P-value	Relapse*		P-value
		Alive	Died		NO	YES	
Age	45	32	12	0.076	31	9	0.708
	≥45	8	9		13	2	
Stage	I-III	5	2	1	1	2	0.328
	IV	38	20		7	0	
Status Before HSCT	PR+NR	20	17	0.027	26	9	0.072
	CR	21	4		20	1	
Prior splenectomy	NO	24	8	0.408	22	6	0.657
	YES	5	4		6	3	
Donor	SIB	8	9	0.179	14	2	0.52
	Haplo	6	1		7	0	
	MUD	12	6		15	3	
Graft	BM	12	7	1	13	5	0.18
	PB	10	6		15	1	
Conditioning include TBI	NO	12	8	0.355	13	5	0.259
	YES	25	8		27	4	
Conditioning intnsity	MAC	17	7	0.709	22	2	1
	RIC	7	4		10	1	
GVHD	NO	8	2	0.688	10	0	1
	YES	18	9		25	2	
Relapse	NO	34	14	0.042	–	–	–
	YES	4	7		–	–	–

*Each group includes only the patients for whom data is available.

CR, complete remission; PR, partial remission; NR, no remission; TBI, total body irradiation; SIB, sibling; Haplo, haploidentical; MUD, matched unrelated donor; UCB, unrelated core blood; MAC, myeloablative conditioning; RIC, reduced intensity conditioning; NMAC, non-myeloablative; BM, bone marrow; PB, peripheral blood; GVHD, graft versus host disease.

There were 53 patients with complete data documenting the use of TBI as a component of the transplant conditioning regimen. The groups had no substantial statistical disparities in prognosis or adverse outcomes. Patients who had TBI as part of their transplant preparation showed higher expected 3-year OS rates and 5-year OS rates compared to those who did not get TBI (71% vs 67%, 71% vs 58%). The group of individuals with TBI exhibited a decreased rate of relapse in comparison to the group without TBI (25% vs 40%). The incidence of GVHD was 66.7% in the TBI group and 83.3% in the non-TBI group. The group of individuals with TBI demonstrated a substantially lower rate of relapse in comparison to the group without TBI (12.9% vs 27.8%). The mortality rate was 24.2% among individuals with TBI and 40% among those without TBI.

**Figure 3:**
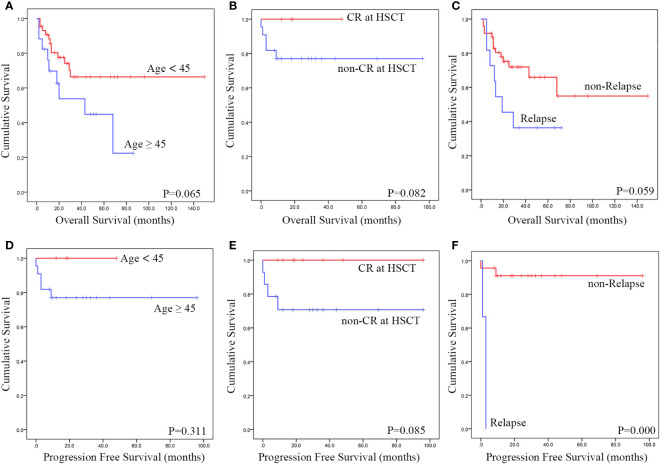
Using data from published cases, a Kaplan-Meier survival analysis was conducted to assess the impact of various risk factors. The findings revealed that Patients under 45 years of age **(A, D)**, transplant status of CR **(B, E)** and relapse after transplantation **(C, F)** exhibited a improved OS and PFS.

We conducted additional studies of patients’ OS and PFS (**Supplementary Table S3**). All patients who were enrolled in the study had OS data available. However, there were challenges in estimating PFS. We attempted to replace PFS nodes with post-transplant progression-free survival time, but only 25 patients reported explicit PFS durations. Prognosis may be affected by factors such as age (**Figures 3A, D**), the state of remission before a transplant (**Figures 3B, E**) and relapse after transplantation (**Figures 3C, F**) when considering the operating system. Before transplantation, attaining CR may affect patients’ PFS, and after the transplant, the recurrence of the disease seems to be associated with a lower PFS (as shown in the Table). Furthermore, the prognosis does not appear to be affected by either splenectomy or the transplant conditioning regimen.

## Discussion

HSTCL is a rare and aggressive type of peripheral T cell lymphoma (PTCL) that most frequently affects young males, with a median age at diagnosis of 32 years. First reported in 1990 (9), HSTCL accounts for < 1% of all PTCL cases and is a rapidly progressive disease that is poorly responsive to chemotherapy (8). An estimated 20% of patients with HSTCL exhibit underlying immunosuppression, and viral infections (including EBV and hepatitis B virus) are rarely reported to be associated with HSTCL onset (10). Greater HSTCL incidence rates among inflammatory bowel disease patients undergoing TNF inhibitor treatment have been reported. Bernstein et al. (11) noted that the risk of this form of lymphoma was elevated among individuals with Crohn’s disease. Kandiel et al. (12) conducted a meta-analysis in which they found that lymphoid malignancy risk was increased four-fold among patients receiving biologics. Ochenrider et al. (13) additionally surveyed reports about 28 HSTCL patients with Crohn’s disease and found that all of these patients underwent azathioprine or thiopurine treatment. At the same time, 79% were treated with the biological infliximab.

HSTCL presents in a manner distinct from other more common lymphomas, with affected patients often exhibiting hepatosplenomegaly and prominent systemic symptoms without substantial lymph node involvement. Routine blood analyses may detect cytopenia without significant bone marrow infiltration (8). Other common presentations include Coombs-negative autoimmune hemolytic anemia and moderately elevated transaminase levels. Mild myeloid cell pathological hematopoiesis may be evident in the bone marrow, and significantly elevated lactate dehydrogenase levels are often detected. Both patients in this report exhibited thrombocytopenia when initially diagnosed, consistent with the fact that over 90% of patients reportedly develop thrombocytopenia that may be indicative of disease progression (14). Case 1 was initially diagnosed with immune thrombocytopenia, and the initial efficacy of hormonal treatment may have been attributable to a partial effect of this therapeutic intervention on lymphoma cells before the onset of hormone resistance.

HSTCL patients exhibit a 5-year OS rate of under 7%, with a median OS interval of just 10 months. Early induction therapy is essential when managing these patients, and while some patients undergo chemotherapeutic treatment, the response duration is generally short (8, 15). Thus, optimal patient outcomes depend on diagnosing patients and planning to perform HSCT early. In patients necessitating radical treatment, high-intensity chemotherapy is recommended, and given the absence of definitive guidelines for first-line chemotherapeutic regimens for these patients, approaches similar to those utilized for PTCL, such as CHOP or ECHOP regimens, can be implemented as initial treatments. In their single-center study of 14 HSTCL patients, Voss et al. (6) found that the median patient age was 36 years, and all patients presented with stage IV disease. Non-CHOP induction therapies were used for most patients, with most receiving ifosfamide-based induction regimens and 8 receiving a non-CHOP regimen, of whom 5 achieved remission and underwent subsequent auto- or allo-HSCT. The prognosis of CHOP regimens was reportedly poor in two studies by Belhadj et al. (14) and Falchook et al. (16). As such, the two patients in the present report were initially treated with non-CHOP induction chemotherapy. In Case 2, the patient responded poorly to a GDP regimen but achieved therapeutic remission after switching to an ICE regimen and currently remains disease-free after bridging transplantation. In Case 1, initial ICE induction chemotherapy led to CR, and the patient remained disease-free after bridging allogeneic HSCT. These outcomes align well with prior evidence that ifosfamide-based regimens are associated with better patient treatment responses.

The value of HSCT as a form of consolidation therapy in HSTCL patients has yet to be established. As such, transplantation has the potential to be curative. Patients may experience better outcomes than those who undergo other forms of disease management. While CR can be achieved following induction therapy in some cases, given the short remission duration in most HSTCL cases, early transplantation is generally recommended. In a study of seven HSTCL patients at the Memorial Sloan-Kettering Cancer Center, of whom six had undergone autologous or allogeneic HSCT, the median OS was 65.5 months (6). In the European Bone Marrow Transplant Lymphoma Working Group Study of 25 HSTCL patients (17), 2/18 of the patients who underwent all-HSCT experienced subsequent relapse compared to 5/7 patients who underwent auto-HSCT. The graft-versus-lymphoma effects associated with allo-HSCT may thus provide benefits to treated patients.

Given that HSTCL primarily affects younger patients and is associated with poor outcomes, this suggests that allo-HSCT should be considered as an option for consolidation treatment in appropriate patients. Our comprehensive evaluation revealed that patients under 45 years who have a complete response at the time of transplantation may experience a more favorable prognosis than older patients. Although the impact of TBI on prognosis was insignificant, patients in the TBI group had improved 3-year and 5-year OS rates compared to those in the non-TBI group. Post-transplant relapse has a significant impact on OS. The prognosis was also not impacted by disease stage at the time of transplantation, indicating that even patients with advanced disease at the time of transplantation may attain benefits from HSCT.

One of the two patients in this report underwent allogeneic HSCT while in CR. In contrast, the other underwent this procedure in a stable disease state, with good efficacy in both instances. Limited data from prior studies suggest that myeloablative preconditioning (MAC) is not associated with any improvements in efficacy relative to reduced intensity conditioning (RIC), suggesting that the graft-versus-lymphoma effect is the primary advantage of allogeneic transplant preconditioning. Treatment regimens incorporating TBI offer advantages for the management of relapsed and refractory lymphatic tumor patients, mainly as they provide a means of more effectively targeting sheltered tumor cells. The treatment regimen employed in this case report was between that of RIC and MAC (18), and these patients achieved deep remission following FACT pretreatment without any significant pretreatment-associated side effects. Patient 2 exhibited splenomegaly on the initial diagnosis, and while splenic enlargement was still evident on PET-CT scanning, the corresponding SUV value was low, and the patient exhibited no cytopenia or evidence of recurrent disease. This patient thus achieved CR, which continues to be accurate as of the most recent follow-up. In another report focused on HSTCL (19), four patients were identified as long-term survivors, with three of these patients having undergone splenectomy alone, including the patient with the longest survival duration of 137 months. Splenectomy may thus represent a viable alternative treatment strategy for patients with disease confined to the spleen who are refractory to chemotherapy or transplantation.

## Conclusion

In conclusion, HSTCL has been associated with a very unfavorable prognosis, highlighting the necessity for further clinical trials to elucidate the significance and ideal timing of allo-HSCT in affected individuals. Younger patients can benefit significantly from allogeneic transportation due to the disease’s tendency for rapid progression and brief remission periods; therefore, it is recommended that this procedure be executed shortly after remission is achieved, with a pretreatment regimen that includes TBI constituting a viable strategy. Even in patients with relapsed and refractory HSTCL, allo-HSCT can provide benefits. Nonetheless, considering that high treatment -related mortality may be evident even in the early phases, the patient’s overall condition should be considered when determining the most suitable transplantation protocol.

## Data availability statement

The original contributions presented in the study are included in the article/**Supplementary Material**. Further inquiries can be directed to the corresponding authors.

## Ethics statement

Written informed consent was obtained from the individual(s), and minor(s)’ legal guardian/next of kin, for the publication of any potentially identifiable images or data included in this article.

## Author contributions

CC: Writing – original draft, Writing – review & editing. FY: Data curation, Investigation, Writing – review & editing. PM: Data curation, Formal analysis, Writing – review & editing. PS: Investigation, Writing – original draft. SQ: Project administration, Writing – original draft, Writing – review & editing.
