# Supplementary material for: Allo-HSCT with TBI-based preconditioning for hepatosplenic T-cell lymphoma: two case reports and systematic review of literature
Source: Front Oncol. 2024 Jan 29;14:1345464. doi: 10.3389/fonc.2024.1345464 (PMC10859473; doi:10.3389/fonc.2024.1345464)
Supplement: Supplementary file 1 [file DataSheet_1.docx]

Figure S1. Search Strategy and flow chart of systematic review.

Searches were done of Pubmed, Embase, and Medline databases (on 6 June, 2022), including conference proceedings. Search terms entered were: (((((Gamma-delta T-cell lymphomas[Title/Abstract])) OR (Hepatosplenic T-cell lymphoma[Title/Abstract])) OR (gammadelta T-cell lymphomas[Title/Abstract])) OR (Hepatosplenic g-d T-Cell Lymphoma[Title/Abstract])) AND (((((((((((((((((Stem Cell Transplantation[Title/Abstract]) OR (Stem Cell Transplantations[Title/Abstract])) OR (Transplantations, Stem Cell[Title/Abstract])) OR (Transplantation, Stem Cell[Title/Abstract])) OR (Cord Blood Stem Cell Transplantation[Title/Abstract])) OR (Stem Cell Transplantation, Placental Blood[Title/Abstract])) OR (Umbilical Cord Blood Stem Cell Transplantation[Title/Abstract])) OR (Placental Blood Stem Cell Transplantation[Title/Abstract])) OR (Stem Cell Transplantation, Cord Blood[Title/Abstract])) OR (Blood Stem Cell Transplantation, Umbilical Cord[Title/Abstract])) OR (Hematopoietic Stem Cell Transplantation[Title/Abstract])) OR (Stem Cell Transplantation, Hematopoietic[Title/Abstract])) OR (Transplantation, Hematopoietic Stem Cell[Title/Abstract])) OR (Peripheral Blood Stem Cell Transplantation[Title/Abstract])) OR (Peripheral Stem Cell Transplantation[Title/Abstract])) OR (Stem Cell Transplantation, Peripheral[Title/Abstract])) OR (Transplantation, Peripheral Stem Cell[Title/Abstract]))

Flow chart of systematic review


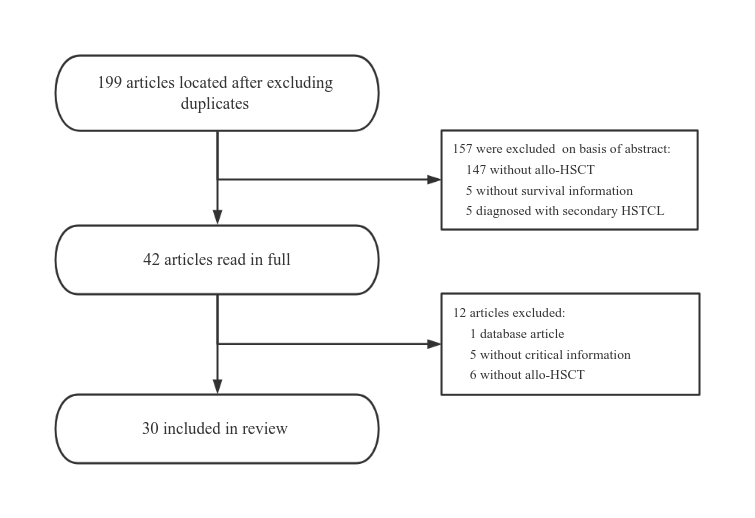


Table S1 Data sheet of reviewed articles.

| Gender | Age | Stage | Status at transplantation | TBI | Outcome | Overall survival (m) | Ref |
| --- | --- | --- | --- | --- | --- | --- | --- |
| M | 20 | IV | PR | No | Alive | 72 | 1 |
| F | 45 | III | PR | No | Died | 43 | 2 |
| F | 38 | IV | NR | Yes | Alive | 44 | 3 |
| F | 17 | IV | NR | Yes | Alive | 32.5 | 3 |
| F | 8 | IV | NR | Yes | Alive | 28 | 3 |
| M | 18 | III | N.A | No | Alive | 10 | 4 |
| M | 23 | IV | CR | Yes | Alive | 36 | 5 |
| M | 46 | IV | CR | No | Alive | 50.5 | 6 |
| M | 51 | IV | CR | Yes | Died | 9.9 | 6 |
| M | 23 | IV | PR | No | Alive | 66 | 6 |
| M | 12 | IV | PR | Yes | Died | 13.1 | 6 |
| M | 19 | IV | PR | Yes | Alive | 149.6 | 6 |
| M | 27 | IV | CR | Yes | Alive | 4.7 | 6 |
| M | 18 | IV | CR | Yes | Alive | 3 | 6 |
| M | 29 | IV | PR | Yes | Alive | 36 | 7 |
| M | 29 | IV | CR | Yes | Alive | 24 | 7 |
| M | 31 | IV | PR | No | Alive | 69 | 8 |
| F | 3 | IV | PR | Yes | Alive | 96 | 9 |
| F | 45 | IV | NR | Yes | Alive | 12 | 10 |
| M | 51 | IV | CR | No | Alive | 48 | 11 |
| F | 20 | IV | N.A | Yes | Died | 5 | 12 |
| F | 53 | IV | CR | Yes | Alive | 19 | 13 |
| M | 48 | IV | PR | No | Alive | 18 | 14 |
| M | 23 | IV | PR | Yes | Died | 19 | 15 |
| M | 24 | IV | CR | Yes | Alive | 9 | 16 |
| F | 17 | IV | PR | Yes | Died | 3 | 17 |
| F | 14 | IV | CR | Yes | Alive | 16 | 18 |
| F | 17 | IV | CR | Yes | Alive | 21 | 19 |
| F | 15 | IV | CR | Yes | Alive | 24 | 20 |
| M | 19 | IV | CR | Yes | Alive | 96 | 20 |
| N.A | N.A | IV | CR | N.A | Alive | 10 | 21 |
| N.A | N.A | IV | PR | N.A | Alive | 27 | 21 |
| N.A | N.A | IV | NR | N.A | Died | 11 | 21 |
| N.A | N.A | IV | CR | N.A | Alive | 12 | 21 |
| F | 17 | IV | PR | N.A | Died | 13 | 22 |
| M | 11 | III | PR | N.A | Alive | 20 | 22 |
| M | 23 | IV | PR | N.A | Died | 8 | 23 |
| F | 18 | IV | N.A | N.A | Alive | 12 | 23 |
| M | 49 | IV | N.A | N.A | Alive | 86 | 24 |
| M | 51 | IV | N.A | N.A | Alive | 7 | 24 |
| M | 19 | IV | NR | N.A | Alive | 12 | 25 |
| N.A | N.A | N.A | CR | No | Alive | 36 | 26 |
| N.A | N.A | N.A | CR | No | Alive | 14 | 26 |
| M | 46 | IV | PR | N.A | Died | 5 | 27 |
| M | 30 | IV | NR | N.A | Died | 30 | 27 |
| M | 31 | IV | NR | N.A | Died | 11 | 28 |
| M | 18 | N | PR | No | Died | 12 | 29 |
| M | 45 | IV | PR | No | Died | 18 | 30 |
| F | 33 | IV | CR | Yes | Alive | 48 | 30 |
| F | 37 | III | CR | Yes | Alive | 20 | 30 |
| M | 47 | IV | PR | No | Died | 68 | 30 |
| M | 36 | III | CR | No | Died | 25 | 30 |
| F | 38 | IV | PR | Yes | Alive | 57 | 30 |
| M | 36 | IV | NR | Yes | Died | 3 | 30 |
| M | 25 | IV | NR | Yes | Died | 29 | 30 |
| M | 59 | IV | PR | No | Died | 2 | 30 |
| F | 38 | I | CR | Yes | Alive | 84 | 30 |
| M | 45 | IV | CR | Yes | Died | 2 | 30 |
| M | 67 | IV | PR | No | Died | 20 | 30 |
| M | 39 | IV | CR | Yes | Alive | 34 | 30 |
| F | 33 | IV | PR | Yes | Alive | 27 | 30 |
| M | 32 | IV | NR | No | Alive | 36 | 30 |
| M | 44 | IV | PR | No | Alive | 34 | 30 |
| M | 59 | IV | CR | No | Died | 11 | 30 |
| F | 54 | IV | PR | No | Alive | 53 | 30 |
| M | 29 | I | CR | Yes | Alive | 28 | Current |
| M | 30 | IV | PR | Yes | Alive | 36 | Current |

1.Yamazaki T, Sawada U, Kura Y,elt. Treatment of high-risk peripheral T-cell lymphomas other than anaplastic large-cell lymphoma with a dose-intensified CHOP regimen followed by high-dose chemotherapy. A single institution study. Acta Haematol. 2006;116(2):90-5. doi: 10.1159/000093637.

2.Bennett M, Matutes E, Gaulard P. Hepatosplenic T cell lymphoma responsive to 2'-deoxycoformycin therapy. Am J Hematol. 2010 Sep;85(9):727-9. doi: 10.1002/ajh.21774.

3.Huang W, Xue S, Zhang Y, Liu F, Tian M, Wang Y, Li F, Lu Y, Wang J. Refractory hepatosplenic T-cell lymphoma was successfully treated with salvage allogeneic hematopoietic stem cell transplantation combined with enhanced myeloablative preconditioning. Ann Hematol. 2023 Jan;102(1):175-180. doi: 10.1007/s00277-022-05017-0. Epub 2022 Nov 10. PMID: 36355191.

4.Kapur LH, Khaled Y, Solh M, Ward D, Chang CC. De novo CD3 negative hepatosplenic T-cell lymphoma: diagnostic challenges and pitfalls. Arch Pathol Lab Med. 2014 Jul;138(7):969-73. doi: 10.5858/arpa.2013-0074-CR. PMID: 24978925.

5.Lolli G, Casadei B, Stefoni V, Argnani L, Bonifazi F, Zinzani PL. Hepatosplenic T-Cell Non-Hodgkin Lymphoma Cured with Tandem Autologous and Allogeneic Stem Cell Transplantation. Chemotherapy. 2022;67(4):253-255. doi: 10.1159/000524891. Epub 2022 May 10. PMID: 35537403.

6.Voss MH, Lunning MA, Maragulia JC, Papadopoulos EB, Goldberg J, Zelenetz AD, Horwitz SM. Intensive induction chemotherapy followed by early high-dose therapy and hematopoietic stem cell transplantation results in improved outcome for patients with hepatosplenic T-cell lymphoma: a single institution experience. Clin Lymphoma Myeloma Leuk. 2013 Feb;13(1):8-14. doi: 10.1016/j.clml.2012.09.002. Epub 2012 Oct 27. PMID: 23107915; PMCID: PMC4056251.

7.Bron D, De Leval L, Michiels S, Wittnebel S; EuroBloodNet for rare diseases. Hepatosplenic T-cell lymphoma: treatment challenges. Curr Opin Oncol. 2021 Sep 1;33(5):406-411. doi: 10.1097/CCO.0000000000000775. PMID: 34409955.

8.Jiang L, Abati AD, Wilson W, Stetler-Stevenson M, Yuan C. Persistent non-neoplastic gammadelta-T cells in cerebrospinal fluid of a patient with hepatosplenic (gammadelta) T cell lymphoma: a case report with 6 years of flow cytometry follow-up. Int J Clin Exp Pathol. 2009 Oct 15;3(1):110-6. PMID: 19918335; PMCID: PMC2776263.

9.Honda T, Yamaoka M, Terao YM, Hasegawa D, Kumamoto T, Takagi M, Yoshida K, Ogawa S, Goto H, Akiyama M. Successful treatment of hepatosplenic T-cell lymphoma with fludarabine, high-dose cytarabine and subsequent unrelated umbilical cord blood transplantation. Int J Hematol. 2022 Jan;115(1):140-145. doi: 10.1007/s12185-021-03229-0. Epub 2021 Sep 30. PMID: 34591292.

10.Okuni M, Yakushijin K, Uehara K, Ichikawa H, Suto H, Hashimoto A, Tanaka Y, Shinzato I, Sakai R, Mizutani Y, Nagao S, Kurata K, Kakiuchi S, Miyata Y, Inui Y, Saito Y, Kawamoto S, Yamamoto K, Ito M, Matsuoka H, Minami H. Successful Bridging Chemotherapy with Gemcitabine, Carboplatin, and Dexamethasone before Unrelated Stem Cell Transplantation for Hepatosplenic T-cell Lymphoma. Intern Med. 2019 Mar 1;58(5):707-712. doi: 10.2169/internalmedicine.1266-18. Epub 2018 Nov 19. PMID: 30449784; PMCID: PMC6443557.

11.Iwaki N, Mochizuki K, Ozaki J, Maeda Y, Kurokawa T. A case of hepatosplenic T-cell lymphoma successfully treated by HLA haploidentical stem cell transplantation. J Clin Exp Hematop. 2020 Jun 20;60(2):55-59. doi: 10.3960/jslrt.20003. Epub 2020 May 13. PMID: 32404572; PMCID: PMC7337272.

12.Jacobs MF, Anderson B, Opipari VP, Mody R. Hepatosplenic αβ T-Cell Lymphoma as Second Malignancy in Young Adult Patient With Previously Undiagnosed Ataxia-Telangiectasia. J Pediatr Hematol Oncol. 2020 Aug;42(6):e463-e465. doi: 10.1097/MPH.0000000000001537. PMID: 31259827; PMCID: PMC6933092.

13.Sumi M, Takeda W, Kaiume H, Kirihara T, Kurihara T, Sato K, Ueki T, Hiroshima Y, Ueno M, Ichikawa N, Kobayashi H. Successful treatment with reduced-intensity cord blood transplant in a patient with relapsed refractory hepatosplenic T-cell lymphoma. Leuk Lymphoma. 2015 Apr;56(4):1140-2. doi: 10.3109/10428194.2014.947613. Epub 2014 Aug 19. PMID: 25065703.

14.Catania G, Zallio F, Monaco F, Corsetti MT, Trincheri N, Bonello L, Mele L, Dallavalle F, Salvi F, Pini M. Successful HLA haploidentical myeloablative stem cell transplantation for aggressive hepatosplenic alpha/beta (αβ) T-cell lymphoma. Leuk Res Rep. 2014 Oct 28;3(2):90-3. doi: 10.1016/j.lrr.2014.09.001. PMID: 25429355; PMCID: PMC4242978.

15.Kawai H, Matsushita H, Ohmachi K, Kojima M, Machida S, Ogawa Y, Kawada H, Nakamura N, Ando K. Four hepatosplenic T-cell lymphoma cases of Japanese patients. Leuk Res Rep. 2015 Dec 15;5:3-6. doi: 10.1016/j.lrr.2015.12.001. PMID: 26870659; PMCID: PMC4711307.

16.Pan H, Huang J, Li JN, Yang L, Wang JY, Wang X, Liu L, Yang ZS, Wang L. Successful second allogeneic stem-cell transplantation from the same sibling donor for a patient with recurrent hepatosplenic gamma-delta (γ/δ) T-cell lymphoma: A case report. Medicine (Baltimore). 2018 Nov;97(44):e12941. doi: 10.1097/MD.0000000000012941. PMID: 30383643; PMCID: PMC6221602.

17.Dhir A, Hill B, Waite ES, Cairo MS, Xavier AC. Pralatrexate-based therapy induced response in an adolescent with refractory hepatosplenic T-cell lymphoma. Pediatr Blood Cancer. 2020 Nov;67(11):e28460. doi: 10.1002/pbc.28460. Epub 2020 Aug 29. PMID: 32860655.

18.Lauhan CR, Schiff D, Gloude N. Successful treatment of hepatosplenic T cell lymphoma in an adolescent with Turner syndrome using ifosfamide, carboplatin, and etoposide followed by allogeneic hematopoietic stem cell transplant. Pediatr Blood Cancer. 2020 Nov;67(11):e28528. doi: 10.1002/pbc.28528. Epub 2020 Aug 9. PMID: 32776434.

19.Schafer E, Chen A, Arceci RJ. Sustained first remission in an adolescent with hepatosplenic T-cell lymphoma treated with T-cell leukemia induction, nucleoside analog-based consolidation, and early hematopoietic stem cell transplant. Pediatr Blood Cancer. 2009 Dec;53(6):1127-9. doi: 10.1002/pbc.22129. PMID: 19479793; PMCID: PMC3095047.

20.McThenia SS, Rawwas J, Oliveira JL, Khan SP, Rodriguez V. Hepatosplenic γδ T-cell lymphoma of two adolescents: Case report and retrospective literature review in children, adolescents, and young adults. Pediatr Transplant. 2018 Aug;22(5):e13213. doi: 10.1111/petr.13213. Epub 2018 Jun 19. PMID: 29921021.

21.Wang Q, Jiang Y, Zhu Q, Duan Y, Chen X, Xu T, Jin Z, Li C, Wu D, Huang H. Clinical features and treatment outcomes of 14 patients with hepatosplenic γ δ T-cell lymphoma. J Cancer Res Clin Oncol. 2021 Nov;147(11):3441-3445. doi: 10.1007/s00432-021-03587-6. Epub 2021 Apr 15. PMID: 33856526.

22.Al Mahmoud R, Weitzman S, Schechter T, Ngan B, Abdelhaleem M, Alexander S. Peripheral T-cell lymphoma in children and adolescents: a single-institution experience. J Pediatr Hematol Oncol. 2012 Nov;34(8):611-6. doi: 10.1097/MPH.0b013e3182707592. PMID: 23042011.

23.Brinkert F, Arrenberg P, Krech T, Grabhorn E, Lohse A, Schramm C. Two Cases of Hepatosplenic T-Cell Lymphoma in Adolescents Treated for Autoimmune Hepatitis. Pediatrics. 2016 Sep;138(3):e20154245. doi: 10.1542/peds.2015-4245. Epub 2016 Aug 11. PMID: 27516526.

24.Yabe M, Medeiros LJ, Daneshbod Y, Davanlou M, Bueso-Ramos CE, Moran EJ, Young KH, Miranda RN. Hepatosplenic T-cell lymphoma arising in patients with immunodysregulatory disorders: a study of 7 patients who did not receive tumor necrosis factor-α inhibitor therapy and literature review. Ann Diagn Pathol. 2017 Feb;26:16-22. doi: 10.1016/j.anndiagpath.2016.10.005. Epub 2016 Oct 18. PMID: 28038706; PMCID: PMC5560101.

25.Cooke CB, Krenacs L, Stetler-Stevenson M, Greiner TC, Raffeld M, Kingma DW, Abruzzo L, Frantz C, Kaviani M, Jaffe ES. Hepatosplenic T-cell lymphoma: a distinct clinicopathologic entity of cytotoxic gamma delta T-cell origin. Blood. 1996 Dec 1;88(11):4265-74. PMID: 8943863.

26.Falchook GS, Vega F, Dang NH, Samaniego F, Rodriguez MA, Champlin RE, Hosing C, Verstovsek S, Pro B. Hepatosplenic gamma-delta T-cell lymphoma: clinicopathological features and treatment. Ann Oncol. 2009 Jun;20(6):1080-5. doi: 10.1093/annonc/mdn751. Epub 2009 Feb 23. PMID: 19237479; PMCID: PMC4092251.

27.Przybylski GK, Wu H, Macon WR, Finan J, Leonard DG, Felgar RE, DiGiuseppe JA, Nowell PC, Swerdlow SH, Kadin ME, Wasik MA, Salhany KE. Hepatosplenic and subcutaneous panniculitis-like gamma/delta T cell lymphomas are derived from different Vdelta subsets of gamma/delta T lymphocytes. J Mol Diagn. 2000 Feb;2(1):11-9. doi: 10.1016/s1525-1578(10)60610-1. PMID: 11272897; PMCID: PMC1906890.

28.Zeidan A, Sham R, Shapiro J, Baratta A, Kouides P. Hepatosplenic T-cell lymphoma in a patient with Crohn's disease who received infliximab therapy. Leuk Lymphoma. 2007 Jul;48(7):1410-3. doi: 10.1080/10428190701345433. PMID: 17613771.

29.A case of hepatosplenic gamma-delta T-cell lymphoma with a transient response to Fludarabine and Alemtuzumab.

30.Tanase A, Schmitz N, Stein H, Boumendil A, Finel H, Castagna L, Blaise D, Milpied N, Sucak G, Sureda A, Thomson K, Vandenberghe E, Vitek A, Dreger P; Lymphoma Working Party of the EBMT. Allogeneic and autologous stem cell transplantation for hepatosplenic T-cell lymphoma: a retrospective study of the EBMT Lymphoma Working Party. Leukemia. 2015 Mar;29(3):686-8. doi: 10.1038/leu.2014.280. Epub 2014 Sep 19. PMID: 25234166.

Table S2 Clinical factors related to GVHD

| Clinical Chracteristics* | | GVHD* | | |
| --- | --- | --- | --- | --- |
|  |  | YES | NO | P-value |
| Conditioning intnsity | MAC | 17 | 5 | 0.217 |
|  | RIC | 5 | 5 |  |
| Graft | BM | 10 | 3 | 1 |
|  | PB | 10 | 4 |  |
| Donor | SIB | 9 | 4 | 0.851 |
|  | Haplo | 5 | 2 |  |
|  | MUD | 11 | 3 |  |

*Each group includes only the patients for whom data is available.

GVHD, graft versus host disease; MAC, myeloablative conditioning; RIC, reduced intensity conditioning; BM, bone marrow; PB, peripheral blood; SIB, sibling; Haplo, haploidentical; MUD, matched unrelated donor

Table S3 K-M survival analysis of clinical factors

| Clinical Chracteristics* | OS | PFS |
| --- | --- | --- |
|  | P-value | P-value |
| Gender | 0.117 | 0.654 |
| Age ≥45 | 0.065 | 0.311 |
| Stage IV | 0.642 | 0.413 |
| Status Before HSCT（CR vs non-CR） | 0.082 | 0.085 |
| Prior splenectomy | 0.602 | 0.495 |
| Donor (Haplo vs Sibiling vs MUD) | 0.402 | 0.259 |
| Graft | 0.702 | 0.231 |
| Conditioning include TBI | 0.465 | 0.827 |
| Conditioning intnsity (MAC vs RIC) | 0.920 | 0.351 |
| GVHD | 0.281 | 0.579 |
| Relapse | 0.059 | <0.01 |

* Each group includes only the patients for whom data is available.

OS, overall survival; PFS progression free survival; CR, complete remission; TBI, total body irradiation; Haplo, haploidentical; MUD, matched unrelated donor; MAC, myeloablative conditioning; RIC, reduced intensity conditioning; GVHD, graft versus host disease;
